# Case of Adipocire

**Published:** 1847-04

**Authors:** 


*Case of Adipocire.*—By the Editor of the New York Annalist.

By the courtesy of our friend, Dr. Blakeman, of Bleecker street, we were invited, a few days since, in company with Drs. Miner, Senr., J. K. Rodgers, A. C. Post, Linsley, F. C. Stewart, Borrowe, Williams, Rockwell, A. E. Hosack, &c., to inspect the body of a Mrs. F., which had been disenterred from a burying ground in Twelfth street, in which it had lain for 17 years. It was that of a very large woman. The coffin in which it had been inclosed was very little affected by its inhumation. The form of the cadaver was perfectly preserved. The extremety of the nose was gone, and the features of the face partially discernable. The arms, which were placed along the sides, had become much compressed by the swelling of the body, and the hands had broken off at the wrists.—The remains of one hand, upon the right side of the abdomen, were distinctly visible, and the forefinger, with its nail, was entire. The rotundity of the breast was perfect—the abdomen flattened, as if by compression against the lid of the coffin. The left foot, like the hands, had separated from the ankle. The rotundity and shape of the thighs was remarkably preserved. The shroud adhered to the body and was not decayed, though discolored. The cadaver presented a greenish hue from mould. with which it was covered, and a fresher coat of pure white, had been deposited upon it since its exposure to the air. The cap was distinct upon the head, and the bow of black ribbon on one side, somewhat faded, remained. By the consent of a son of the deceased, who was in possession of the body, a piece about a foot square was cut out of the abdominal walls, by Dr. Post, so as to expose the cavity. The knife passed easily through their substance, which was found to consist of a yellowish cheesy substance. The thin layer of abdominal muscles, could be discriminated, similarly converted, beneath the adipose layer, which was very thick. The inner surface of the abdominal cavity was smooth. The viscera, and a large lump of adipocire, lay at the bottom. The diaphragm was distinctly seen. Below it, and towards the left, much shrunken and condensed, and having the consistence, somewhat, of lung, lay the liver. The stomach, having its natural form and a macerated appearance, was distinctly visible, and some portions of the colon, collapsed and membranous, were discoverable. This was all that could be seen within the abdominal cavity, through the aperture that was made. The exsected piece of abdominal wall was then replaced. An odor closely resembling that of gum ammoniac, exhaled from the body. We learn that the soil in which the coffin was interred, was sandy, and not moist; and that two bodies of children buried over it, and others in the vicinity, were in a state of complete disorganization. Some finger-bones, lying loose in the coffin were taken up, and found so much softened as to be cut easily, but not altered in form. The shavings which the coffin contained, were blackened, but otherwise, unaltered. The subject was 69 years of age, very fat, and weighed 170 pounds. The disease of which she died has escaped our reccollection. The conversion of the human body into ADIPOCIRE, though rare among us, has been not unfrequently met with. A body in this state of preservation was exhibited some time ago, at one of our public museums, coming, we believe from Canada. On the occasion, of removing a very great number of human bodies from the ancient burying-place of the Innocents, at Paris, facts of this nature were observed in the most striking manner, and the celebrated Fourcroy read a memior on the subject in the year 1789, to the Royal Academy of Sciences. The adipocirous bodies were found in common graves or repositories, cavities 30 feet by 20, containing each from 1000 to 1500 bodies.—The first opened, had been closed for 15 years. The bodies were flattened, as if from strong compression ; the linen was slightly adherent to them ; and with the form of the different regions, exhibited, on removing the linen, nothing but irregular masses of a soft ductile matter, of a grey-white color. The bones had no solidity, but broke by any sudden pressure. The white substance, which resembled cheese, yielded to the touch, and became soft when rubbed for a time between the fingers. Contrary to what had occurred in the subject under consideration, the abdominal had disappeared, and scarcely any trace of viscera was observed in the almost obliterated cavity. All the viscera were confounded together, and for the most part, no traces of them were left. The same was true of the contents of the thorax : nothing was seen in either place but some parcels of the fatty matter. The brain was constantly found contracted in bulk, black at the surface, and absolutely changed like the other organs. The fatty matter had undergone various modifications, according to the length of time the bodies had been deposited. Adipocire, according to Fourcroy, consists in a concrete oily, or waxy substance, approaching most nearly to spermaceti, which, in combination with ammonia, forms the animal soap into which the dead bodies are thus converted. Adipocire may be obtained artificially. Chevreul found it composed of a small quantity of ammonia, potash and lime united to margarine, and to a very little of another fatty matter, different from that. Home & Brande (Philosophical Transact., 1843,) believed that adipocire is formed by an incipent and incomplete putrefaction. M. H., æt. 44, was buried May, 1790, in a very damp grave, the tide rising into it, and in 1811 the body was, with some others, buried near it, converted into adipocire. Ambergris contains nearly half its weight of adipocire, and the fatty matters discharged from the human bowels, or found in them, are probably of the same nature. The following is an instance, probably, of the same kind :

In 1796, the tomb of Lady Kilsyth, and her infant child, who had been smothered by the falling of a house in Flanders, in 1717, embowelled and embalmed, and subsequently buried in Scotland, was opened by some reckless young men. With astonishment and consternation, they saw the bodies of Lady Kilsyth and her child, as perfect as at the hour they were entombed. "I saw the body of Lady K.," said a clergyman of the parish, "soon after the coffin was opened ; it was quite entire. Every feature, and every limb was as full, nay, the very shroud was as clear and fresh, and the color of the ribbons as bright, as the day they were lodged in the tomb. The child lay at her knee ; his features were as composed as if he had been only asleep ; his color was as fresh, and his flesh as plump and full, as in the perfect glow of health ; the smile of infancy and innocence sat on his lips. He was only a few months old. The body of Lady K., was equally well preserved, and at a little distance, and by a feeble light, it would not have been easy to distinguish whether she was dead or alive. The body had been preserved in some liquid resembling brandy, which had lost all characteristics, and had tinged the body of a copper hue. After many months, the bodies were as firm and compact as at first, and though pressed with the finger, did not yield to the touch. Some medical gentlemen made a small incision into the arm of the infant: the substance of the body was quite firm."

Making all due allowance for the exaggerations of the writer, this was probably a case of conversion into adipocire. The tomb of Edward I., who died 7th July, 1307, was opened after the lapse of 463 years. The body was not decayed; the flesh on the face was a little wasted, but not putrid. That of Canute the Dane, who got possession of England in 1017, was found very fresh in 1766. The body of Bonaparte was, when the coffin was opened, in a state of remarkable preservation. In 1569, three Roman soldiers, in the dress of their country, fully equipped with warlike instruments, were dug out of a peat moss of great extent, called Kazey Moss.—After the lapse of 1500 years, they were quite fresh and plump.—*N. Y. Annalist.*

